# Gerald Loewi: A Major Contributor to the New Era of Rheumatology Thinking

**DOI:** 10.5041/RMMJ.10280

**Published:** 2017-01-30

**Authors:** Michael Denman

**Affiliations:** Former Head MRC Connective Tissue Diseases Unit, Clinical Research Centre, Harrow, UK; and Emeritus Consultant Physician, Imperial College London, UK

**Keywords:** Gerald Loewi, research, rheumatology

## Abstract

Dr Gerald Loewi was Senior Research Scientist and Consultant Pathologist at the Medical Research Council (MRC) Rheumatism Research Unit at Taplow, England and subsequently the MRC Clinical Research Centre, Harrow, England. An immunologist with a background in pathology, he made major contributions to our understanding of the immunopathology of rheumatoid arthritis and other rheumatic disorders. With his colleagues, he developed a more sophisticated concept of what were initially thought to be primary autoimmune or degenerative diseases but are now recognized as much more complex disease processes. He was one of the first to initiate close collaboration between clinicians and scientists in rheumatology research and practice.

## INTRODUCTION

Much of modern British rheumatology research originated in an academically remote setting far from established centers of national scholarship. In the First World War the Duchess of Connaught Hospital was established in the grounds of Lord Astor’s country estate to serve as a military hospital for Canadian servicemen. Abandoned after the war, it was rebuilt at the start of the Second World War as the Canadian Red Cross Hospital, also for the care of Canadian servicemen. One can reflect on the health economics of the time; the annual rent paid to Lord Astor in those altruistic days was one US dollar. After the Second World War the Canadian government presented the hospital to the British nation, and it was incorporated into the newly established National Health Service. Initially a general hospital, in 1947 the now renamed Canadian Red Cross Memorial Hospital incorporated the Medical Research Council (MRC)’s newly established Special Unit for Juvenile Rheumatism, which later expanded to cover rheumatic diseases at all ages. The rural surroundings were idyllic. However, the Unit’s accommodating wards and laboratories were built at a time of severe national austerity and were not very dissimilar from Ma’abarot of the same epoch in Israel.

## THE RHEUMATISM RESEARCH UNIT, TAPLOW

It was against this challenging background that the rheumatology unit’s first Director, Professor Eric Bywaters, established a clinical research unit whose research activities spanned the entire spectrum of clinical care and scientific research. Clinical care included treatment trials of anti-rheumatic drugs and physical support such as physiotherapy and splint design. Scientific research covered joint structure and function as well as basic issues in inflammation and immunology relating to the nature of rheumatic diseases. The most pressing clinical problem of the time was rheumatic fever and associated carditis. The concept of collaboration between clinicians and basic scientists in rheumatology research is now taken for granted; at that time this was a novel concept implemented only in the USA and a few European centers still struggling to recover from the Second World War. Taplow’s geographical isolation was not an obstacle; Eric Bywaters was joined by an enthusiastic team of colleagues from the United Kingdom. He also attracted a constant stream of visiting workers both from within Britain and from the British Commonwealth, the USA and many other countries, including Israel. The unit’s staff also collaborated closely with colleagues in British universities and other centers. Gerald Loewi joined the staff soon after the unit was established. His colleagues included the immunologists Leonard Glynn and John Holborow, the chemist John Scott, and the rheumatologist Barbara Ansell.

## GERALD LOEWI’S BACKGROUND

**Figure 1:**
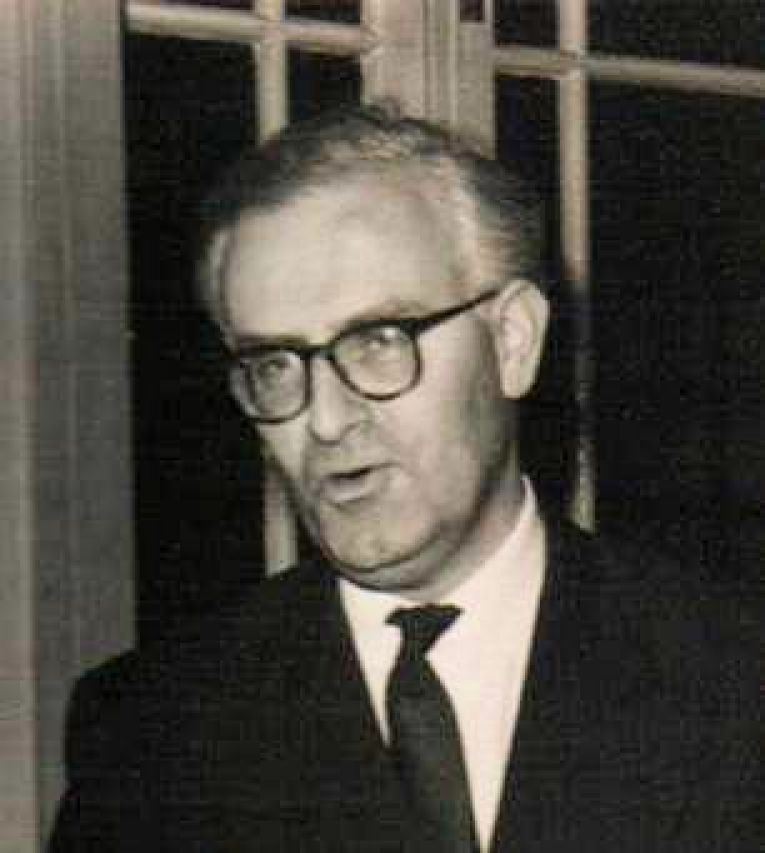
Gerald Loewi.

Gerald Loewi (Figure 1) studied medicine at Oxford University and University College Hospital, London, and his immediate post-graduate training and experience was in pathology. However, he had broadly based scientific interests and a lively curiosity. These were essential intellectual qualities for a clinical scientist seeking a way forward in rheumatology, a field where clinical diagnosis was based on archaic terminology and the route toward understanding the basis of these diseases was similarly obscure. His adolescent experience as a refugee from Nazi Germany, his time as a student in a very traditional English public school and universities, and the inevitable official distrust of him as a perceived alien in the early stages of the Second World War affected Gerald Loewi emotionally and intellectually. Happily, the informal scientific atmosphere and the broad mix of his colleagues’ backgrounds largely dispelled these shadows. In compensation, his background gave him a valuable advantage in that he could easily collaborate with colleagues in a wide range of European and other countries. It also helped him to encourage young, aspiring colleagues, many of whom have had very distinguished careers, including election as Fellows to the Royal Society.

Subsequently in 1969 he moved to the Division of Immunological Medicine at the MRC’s newly established Clinical Research Centre in Harrow, of which he was a member until his untimely death in 1977. The intellectual environment there was similar to that of Taplow but on a much bigger scale.

His main cultural interests were German classical literature and in particular the works of Goethe and Schiller. His interest in philosophy centered especially on the influence of Greco-Roman and medieval thought, and in particular Maimonides, on contemporary scientific thinking. His musical interests centered mainly on opera and eighteenth- and nineteenth-century composers, in particular Mozart, Beethoven, and Schubert. Similarly, his interest in art covered the period ending in Impressionism, but he had rather less interest in what was “contemporary art” in his lifetime.

## GERALD LOEWI’S CONTRIBUTIONS AT TAPLOW

Since Loewi’s death rheumatology research has developed enormously, partly because of advances in underlying basic science, especially genetics and immunology, and partly because of vastly improved clinical investigative techniques, notably imaging and biostatistics. However, it is interesting to reflect that many of the concepts which preoccupied Gerald Loewi and his colleagues are still central to present-day theorizing and speculation. The rationale for setting up the Juvenile Rheumatism Unit in the first place is now clinically irrelevant in most of the world, but the same scientific and clinical management questions continue to preoccupy rheumatologists despite the change in the spectrum of rheumatic diseases. Rheumatic fever has largely receded as an issue, but infection remains an important issue. While rheumatologists speculated on genetic and immunological factors which determine susceptibility to rheumatic fever after streptococcal infection, streptococci quietly left the rheumatology stage. But this was not wasted effort; our increasing realization of the importance and complexity of the microbiota and pandemics of novel infections have ensured the return of micro-organisms to rheumatology’s center stage.

Gerald Loewi and his colleagues were largely preoccupied with the broader issues of infection and the pathogenesis of rheumatic diseases in general and not just rheumatic fever. Indeed, when Leonard Glynn and John Holborow summarized the approach of the Taplow group to these issues, they were careful to name their seminal book *Autoimmunity and Disease*, not *Autoimmune Diseases*.^1^

Gerald Loewi’s personal contributions were largely centered on characterizing the cells participating in inflammatory synovitis. Hitherto these cells had been termed “mononuclear,” but the histological techniques of the day only permitted broad differentiation between “lymphocytes” and “macrophages”. In part he and his colleagues used experimental models of joint inflammation.^2^–^4^ But in addition and more importantly they analyzed cells in effusions and synovial membranes from patients with inflammatory arthritis using immunofluorescence techniques and electron microscopy.^5^–^7^ Their findings helped to identify the key role of T and B lymphocytes in the pathogenesis of synovitis in rheumatoid arthritis and other rheumatic diseases, thereby marking an innovative transition from descriptive to analytical immunopathology in rheumatology research. Gerald Loewi also explored the functions of the infiltrating cells. His collaborative studies with Ian MacLennan provided important insight on the cytotoxic effects on target cells of mononuclear cells isolated from synovial effusions either alone or in combination with specific antibodies.^8^,^9^ As a possible lead to the involvement of virus infection in the etiology of rheumatoid arthritis patients, Bracha Zisman (Rager) and Gerald Loewi analyzed the fate of vesicular stomatitis virus in white blood cells from such patients.^10^

The Taplow investigators were early to appreciate that what were traditionally termed “degenerative joint diseases” or “osteoarthritis” are in reality attributable to much more complex processes than “ageing” alone. Their biochemist colleague John Scott’s main field of interest concerned cartilage and collagen structure and function. His theoretical and technical expertise greatly helped his colleagues to explore the underlying causes of this group of disorders. It is now accepted that all structural joint components are involved in the degenerative process. Gerald Loewi and his collaborators made two fundamental observations supporting this transition in ideas. With Helen Muir, he showed that the major joint component, chondromucoprotein, is antigenic and thereby capable of inciting an auto-immune response.^11^ With Leonard Glynn and Jack Dorling, he made comparable observations relating to the potential auto-antigenicity of degenerating collagen fibers.^12^

There were limited means then available for exploring the genetic basis for the familial link with many rheumatic diseases suggested by contemporary population surveys in children and adults. This was a particular interest of Gerald Loewi’s colleagues, Eric Bywaters and Barbara Ansell. Blood group secretor status was one genetic marker which was available, and Gerald Loewi and John Holborow contributed to the unit’s exploration of this approach.

## GERALD LOEWI’S CONTRIBUTIONS AT THE MRC CLINICAL RESEARCH CENTRE

Gerald Loewi’s final contributions were made in a very different environment. The MRC’s Clinical Research Centre at Northwick Park Hospital, Harrow, was established with the aim of encouraging and supporting collaboration between scientists and clinicians interested in a wide range of diseases and not rheumatology alone. His versatile intellect and expertise were ideally suited for this new and much more open-ended venture. With David Webster, he made novel observations in patients with hypogammaglobulinemia, including the response of the associated polyarthritis to gamma globulin replacement.^13^ He also collaborated closely with the gastroenterology department, which had a particular interest in inflammatory bowel disease, a problem with well-recognized relevance to rheumatology. Gerald Loewi’s contributions included a study with Tony Segal which identified disordered neutrophil function in patients with Crohn’s disease.^14^

## Abbreviations

MRC: Medical Research Council
